# Correlation Analysis of Acute Psychotic Symptom Domain Severity and Fractional Anisotropy of Brain White Matter Tracts in Schizophrenia

**DOI:** 10.17691/stm2025.17.4.01

**Published:** 2025-08-29

**Authors:** V.L. Ushakov, N.V. Zakharova, L.V. Bravve, G.Sh. Mamedova, S.I. Kartashov, G.P. Kostyuk

**Affiliations:** PhD, Associate Professor, Leading Researcher, Institute for Advanced Brain Studies; Institute for Advanced Brain Research, Lomonosov Moscow State University, 27/1 Lomonosov Prospect, Moscow, 119192, Russia; Leading Researcher; Alekseev Psychiatric Clinical Hospital No.1, Moscow Department of Health, 2 Zagorodnoe Shosse, Moscow, 117152, Russia; Senior Researcher; National Research Nuclear University MEPhI, 31 Kashirskoe Shosse, Moscow, 115409, Russia; MD, PhD, Leading Expert, International Scientific and Educational Center of Neuropsychiatry; Samara State Medical University, 89 Chapayevskaya St., Samara, 443099, Russia; Leading Researcher; V.M. Bekhterev National Medical Research Center for Psychiatry and Neurology, Ministry of Health of the Russian Federation, 3 Bekhtereva St., Saint Petersburg, 192019, Russia; Junior Researcher, Laboratory of Fundamental Research Methods, Moscow Research and Clinical Center for Neuropsychiatry; Alekseev Psychiatric Clinical Hospital No.1, Moscow Department of Health, 2 Zagorodnoe Shosse, Moscow, 117152, Russia; Junior Researcher, Laboratory of Fundamental Research Methods, Moscow Research and Clinical Center for Neuropsychiatry; Alekseev Psychiatric Clinical Hospital No.1, Moscow Department of Health, 2 Zagorodnoe Shosse, Moscow, 117152, Russia; Research Engineer; National Research Center “Kurchatov Institute”, 1 Akademika Kurchatova Square, Moscow, 123182, Russia; MD, DSc, Professor, Chief Physician; Alekseev Psychiatric Clinical Hospital No.1, Moscow Department of Health, 2 Zagorodnoe Shosse, Moscow, 117152, Russia

**Keywords:** schizophrenia, hallucinations, connectome, generalized fractional anisotropy, white matter

## Abstract

Brain white matter tracts (connectomes) have been analyzed in patients with schizophrenia whose condition was determined in the paradigm of dimensional approach.

**The aim of the investigation** is to study the connectivity of brain regions depending on the severity of the psychosis clinical picture in schizophrenia.

**Materials and Methods:**

46 patients (22 women and 24 men, average age — 26.5±5.3 years) with the diagnosis of schizophrenia have been examined in the period of the remission onset after a first psychotic episode. The condition severity was determined by assessing scores on the following psychometric scales: PANSS, CRDPSS, BFCRS, NSA-4, FAB. Diffusion-tensor magnetic resonance imaging of the brain was performed using 3Т MRI Magnetom Verio (Siemens Healthineers, Germany). Significant connections between the indicators of generalized fractional anisotropy of the brain pathways and the severity of the psychosis clinical picture were calculated based on the Spearman’s rank correlation coefficient.

**Results:**

Specific structural features of the brain connectome correlating with symptom severity were identified for each symptom domain. Additionally, tracts in which changes were associated with the severity of several symptom domains simultaneously, were visualized. Alterations in the tracts of the right frontal parietal and parolfactory cingulum correlate with the severity of hallucinatory, negative, and catatonic symptoms. Changes in the tracts of the left frontal parahippocampal cingulum correlate negatively with the severity of hallucination and delusion, while changes in the right frontal parahippocampal cingulum correlate with the severity of delusion and catatonia. In cases of severe hallucinations, delusional disorders, and disorganization, the most significant changes are manifested in the tract structures of the left and right fornix. Significant changes in the pathways of the corpus callosum correlate with the intensity of catatonic symptoms and negative symptomatology. Manifestation severity of various domains of psychosis is associated with differences in structural organization of the brain tracts.

**Conclusion:**

There have been received new data on possible differential involvement of the brain structures in the pathogenesis of various schizophrenia manifestations such as hallucinations, delusions, disorganization phenomena, catatonia, and negative disorders, which may be considered as objective neurophysiological markers of the given disease.

## Introduction

The key links of pathogenesis of schizophrenia and other primary psychotic disorders are alterations in the structure and functions of certain brain regions, whose importance is summarized in several meta-analyses based on neuroimaging studies [1–3].

Symptoms of schizophrenia may be combined in domains (dimensions), which are present in various forms of psychotic disorders and also exist in the same patient in different qualitative and/or quantitative combinations [4]. Some clinical domains include positive disorders or distortion of reality perception (delusions and hallucinations), negative disorders (abulia/apathy, anhedonia, asociality, and reduction of emotion expressiveness), psychomotor disturbances (in the form of catatonic phenomena) as well as cognitive and affective disorders and disorganization phenomena (including formal thought disorder).

Despite the ongoing discussions concerning the number of obligatory dimensions (i.e. two- [5], three- [6], five- [7], and multifactorial characteristics, including also social and psychological aspects [8] of studying schizophrenia), dimensional unification of symptoms seems to be most adequate for solving research tasks [9], for example, for studying specific features of connectivity and brain white matter integrity between separate zones.

From the point of view of structural and functional changes of the brain as a substrate of disease development, aberrations of structural connectome determining the probable connections of brain neural networks and, consequently, the degree of neural network cooperation in cognitive processes are of special interest [10]. The architecture of the structural connectome may be assessed using diffusion-tensor magnetic resonance tractography, measuring characteristics of fractional anisotropy of water molecule diffusion in the brain volume [11].

The analysis of the current literature, devoted to the neuroimaging schizophrenia studies, including tractography, has shown that these investigations are usually carried out in two directions. One of them includes searching for specific changes in the structure and functioning of brain neural networks at different stages of the disease progression. Thus, aberrations have been found in the structural connectome of the white matter characteristic, for example, for various stages of schizophrenia — from prodromal or the stage of a high risk of psychosis, to the stage of remission formation after psychosis [12, 13]. The second direction consists in determining specific features in the structural and functional organization of the brain neural networks engaged in the development of some syndromes of schizophrenia. One of the most reproducible results [1, 3, 14–16] is revealing deviations in the frontal and temporoparietal regions, including aberrations not only of the tracts (arcuate tract, corpus callosum, superior and inferior longitudinal tract) but also of morphometric (anatomical) and functional brain characteristics in auditory verbal hallucinations (for example, ERC “VOICE” project [17]). It has been also noted that peculiarities in the brain structures observed in schizophrenia may also be encountered in various combinations and intensity degree in patients with non-psychotic disorders and in individuals from the healthy control group. Partly, it is traced in a vast heterogeneity of the psychosis clinical picture mediated by heterogeneity of the brain structural organization and in the possibility of symptom persistence with continuity of the degree and its intensity from extremely severe forms to the persistence at the subthreshold level [18–19].

When searching for neuroimaging markers of schizophrenia, it is appropriate to build a research paradigm taking into consideration not only dimensional disease structure but also continual severity heterogeneity of symptoms/groups of symptoms inside the dimensional structure, as it is reasonable to suppose that there exist highly specific neurobiological markers with high diagnostic or prognostic value for each domain (dimension).

In this vein, Bopp et al. [20] performed their investigation, in which they conducted correlation analysis of tract fractional anisotropy (FA) and the severity of psychopathological domains comparing the results of diffusion-tensor imaging (DTI) of 26 patients with schizophrenia and 26 participants from the healthy control group. The authors have detected changes in FA in nine tracts of the white matter, which strongly correlated with some psychopathologic domains measured on the SAPS/SANS scales (Scale for the Assessment of Positive Symptoms/Scale for the Assessment of Negative Symptoms). Severity of hallucination symptom positively correlated with FA indicator in the left uncinate fasciculus and left corticospinal tract. Dissociative disorder factor (passivity phenomenon) showed positive correlation with FA in the tract of the right anterior thalamocortical radiations. The severity of the thought disorganization dimension correlated negatively with the FA value in the right cingulum. The degree of negative disorders directly correlated with FA changes in the right anterior thalamocortical radiations and correlated inversely with the right ventral cingulum bundle [20].

According to the hypothesis formulated by us, there is a correlation between the severity of separate groups of symptom domains and changes in the structural organization of the white matter tracts connecting brain regions, which are pathogenetically significant for these domains. To verify this hypothesis, the same patient sample underwent ranking with identification of continual heterogeneity in terms of severity degree of schizophrenia symptom domains.

**The aim of the investigation** was the correlation analysis between the indicators of generalized fractional anisotropy (GFA) of the brain white matter tracts and continually heterogeneous symptom severity across the main psychopathological domains, including hallucinations, delusions, disorganization phenomena, catatonia, and negative disorders.

## Materials and Methods

### Patients

We examined 46 patients (22 women and 24 men; average age — 26.5±5.3 years) admitted to the psychiatric intensive care units of the Alekseev Psychiatric Clinical Hospital No.1 in 2018–2019. Written informed consent was received from all patients after a full description of the examination procedures in compliance with the Declaration of Helsinki (2024). The results of the present study are part of the research program “Molecular and neurophysiological markers of endocrine diseases” carried out at the Alekseev Psychiatric Clinical Hospital No.1 and approved by the Independent Interdisciplinary Committee on Ethical Expertise of Clinical Research of July 14, 2017 (Protocol No.12).

The patients were selected according to the following inclusion criteria: age from 21 to 35 years, the condition meeting schizophrenia criteria (F20) in ICD-10 (International Classification of Diseases) and DSM-5 (Diagnostic and Statistical Manual of Mental Disorders), right-handedness, critical reports on patient’s own condition with preservation of memory about psychotic symptoms, informed consent for participation in the study. Exclusion criteria included schizoaffective and affective disorders, organic brain diseases, severe somatic and/or neurological states potentially influencing physiology or brain structure, signs of psychoactive substance abuse, and general contraindications to magnetic resonance imaging (MRI).

Clinical examination was conducted by two experienced psychiatrists involving all necessary data: questioning of relatives, analysis of medical cards, results of physical and laboratory tests, and so on.

The severity of schizophrenia symptom domains was ranked by the results of clinical interview and after the assessment of patient conditions using psychometric tools, because the clinical picture and dynamics of psychotic disorders seemed to meet the schizophrenia criteria but still demonstrated different psychopathological domains of various severity degree. Thus, prominence of hallucinations in patients varied from separate auditory artefacts to imperative or threatening voices. Delusional syndrome in some cases manifested itself by persecutory ideas with the plot from everyday life (poisoning, jealousy, damage), while in other cases it reflected terrible distortion of thinking with fantastic plots (messianism, supernatural forces or the ability to “build intergalactic fractals”). Besides, patients in acute psychosis were not equally available to contact due to catatonic symptomatology or transient loss of cognitive functions.

Clinical interview was supplemented by the following diagnostic tools: Positive and Negative Syndrome Scale (PANSS); Clinician-Rated Dimensions of Psychosis Symptom Severity (CRDPSS) installed in DSM-5; 4-Item Negative Symptom Assessment (NSA-4); Bush–Francis Catatonia Rating Scale (BFCRS); Frontal Assessment Battery (FAB).

The model of continual hallucination heterogeneity was built based on the scores of PANSS-Р3 and CRDPSS-hal criteria. Auditory hallucinations have been identified among the patients of the tested sample (disorders of other modality perception were not detected), with a severe form (PANSS-Р3 — 5–6 points, CRDPSS-hal — 3–4 points) being noted in 17 patients, moderate (PANSS-Р3 — 4 points, CRDPSS-hal — 2 points) in 14, while presence of hallucinations in the remaining 15 patients was uncertain (PANSS-P — 2–3 points, CRDPSS-hal — 1 point). Such distribution is in line with epidemiological data on the prevalence of hallucinations among 60–80% patients with schizophrenia [21] and with their heterogeneous degree of manifestation — from elementary acoustic artefacts to complex sentences with semantic content such as “voices inside my head” with imperative or threatening character in the most severe degree [21]. In this work, the authors studied the correlation of the GFA indicator with the points on the CRDPSS scale.

Delusion severity was ranked based on the points of PANSS-P1 and CRDPSS-delusion. Delusional disorders were distributed in the following way: 35 patients were diagnosed with severe and moderate forms having several systematized stable ideas with fanciful plots leading to disadaptive behavior (PANSS-Р1 — 4–6 points, CRDPSS-del — 3–4 points); 11 patients had weak delusional disorders (PANSS-Р1 — 2–3 points and CRDPSS-del — 0–2 points) or the disorders were determined at a subthreshold level (one non-systematized idea with a plot from everyday life). Such distribution is also in line with the data of delusional disorder epidemiology in schizophrenia, according to which this domain was encountered in 70% of patients with schizophrenia, and is one of the key diagnostic criteria [22]. In this work, the correlation of the GFA indicator with the score on PANSS-Р1 was studied.

Severity of thought disorganization was determined in compliance with the points of PANSS-P2, CRDPSS-dezorg and using the Frontal Assessment Battery (FAB) tests. Severe events of disorganization (PANSS-Р2 — 5–7 points, CRDPSS-dez — 3–4 points, FAB <12 points) were found in 22 patients; in the rest 24 cases, there was noted the continuum from the mean degree of prominence to the threshold values, which is within the widest range of the diagnostic share for this domain given in the literature, i.e. from 5 to 91% of psychoses [23]. The total score of the tests conducted on the FAB scale was chosen for the correlation analysis with the GFA indicator.

The continuum of catatonic psychomotor abnormalities were built based on the CRDPSS-psychomotor criteria and the sum of the BFCRS points. The catatonia diagnosing has some specific aspects. It has been established that clinical conversation helps diagnose catatonia in 5–10% of patients [24], whereas the assessment of the status with psychometric tools increases this value to 20–43% [25]. This remark is also applicable to the results of the present study. Severe symptoms of catatonia (PANSS-G5 — 5–7 points, CRDPSS-psychomotor >3 points, BFCRS >10 points) were revealed in 16 patients; in other 30 cases we observed single episodic phenomena at the subthreshold level. In this work, the correlation of the GFA indicator with the total score of the BFCRS tests was studied.

Prominence of negative symptoms was evaluated using objective data obtained from the people closest to the patients and from medical records with one-year depth retrospection to exclude phenomenological intersections with depressive symptomatology or neuroleptic phenomena. The results of diagnosis were also compared by three criteria: the total score on PANSS-N, the CRDPSS-negative dimension, and NSA-4 questionnaire. According to the results of the two large epidemiological cross-sectional studies [26, 27], at least one negative symptom is found in 50% of observations. Our data demonstrate that moderate severity of negative symptomatology was identified in 22% of patients (PANSS-N total score >30 points, CRDPSS-negative >3 points, NSA-4 >15 points) taking into account the retrospective assessment of the state as deep as a year preceding the manifestation of real psychosis. In other cases, negative disorders were presented at the mild or uncertain level. The total score of the tests on the NSA-4 was taken for the correlation analysis with the GFA indicator.

Thus, five ranked samples based on the same group of patients, which reflected continual heterogeneity of the respective dimension severity, i.e. hallucination, delusion, thought disorganization, catatonia, negative symptoms, have been formed for correlation analysis.

### MRI scanning

#### Equipment

The study was conducted at the National Research Center “Kurchatov Institute” using Magnetom Verio MRI scanner (Siemens Healthineers, Germany) with 3.0 T magnetic field strength. A set of data was obtained for the analysis: structural 3D T1-weighted images in the sagittal plane (slice number — 176, repetition time — 2530 ms, echo time — 3.31 ms, slice thickness — 1 mm, deviation angle — 7°, inversion time — 1200 ms, field of view (FOV) — 256×256 mm^2^), two sets of data of diffusion MRI with 64 directions of diffusion encoding gradients with opposite directions of phase encoding (b-factor 1500 s/mm^2^, number of slices — 64, repetition time — 13,700 ms, echo time — 101 ms, slice thickness — 2 mm, flip angle — 90°, FOV — 240×240 mm^2^).

#### Preprocessing of diffusion MRI data

Diffusion data were converted from DICOM to NIfTI format. Next, correction of diffusion data distortion artifacts was done by means of topup module in the FSL program (https://fsl.fmrib.ox.ac.uk/fsl/fslwiki/FSL): assessing distortions in two sets of data, building a map of magnetic field non-uniformity inside the scanner, and thereafter data were corrected and smoothed according to the obtained maps. Minimal length of the reconstructed tracts was 10 voxels (20 mm).

Based on the method of local connectomes, we analyzed the GFA indicator, which solves the problem of tract intersection better than FA [28]. GFA characterizes the generalized difference of the medium in each voxel: degree of myelination, density and thickness of the axonal pathways [29]. While the majority of the human structural connectome analyses are based on the search for global connectivity patterns on both ends of the anatomical pathways selected as zones of interest, the local connectome analysis, called connectometry, traces local patterns of connectivity along the fiber paths themselves. This allows for the identification of the pathway subcomponents, which express important associations with the tested variable [30].

#### Statistical processing

The corrected diffusion data were normalized in the DSI Studio program (http://dsi-studio.labsolver.org) to the MNI space using the q-space diffeomorphic reconstruction (QSDR) to obtain the spin distribution function. The artifacts caused by the head movement and eddy current induced by the change of the value and polarity of gradient fields inside the scanner were additionally corrected. All the corrected diffusion data were included into the connectometric base for the correlation analysis of the derived parameter of the diffusion MRI data (GFA coefficient) and the degree of symptomatology prominence.

To analyze statistically the interrelation of tract specific features with psychopathological manifestations, Spearman’s non-parametric correlation with a threshold of the t-criterion equal to 2.5 (the significance threshold value of 0.05) was used. Application of the Spearman’s rank correlation coefficient is explained by the absence of normal distribution of the dependent values (Kolmogorov–Smirnov test) and a non-linear monotonicity in the relationship of the correlated variables (Pearson correlation coefficient assesses linear relationships). As the result of the statistical analysis, regions (voxels), in which the GFA value correlated positively or negatively with the continual heterogeneity of the symptom severity, were localized. Further, their attribution to the specific tractographic pathways was determined using the HCP-842 atlas (http://brain.labsolver.org/diffusion-mri-templates/hcp-842-hcp-1021). An additional correction for multiple comparisons (false discovery rate (FDR) or expected ratio of erroneous discoveries) were taken into consideration for each comparison of clinical symptomatology. To remove false relationships, 4-iteration correction considering topological brain specificities was performed. According to the method of local connectometry of the DSI Studio program [30], 4000 random permutations in the independent group variables (scores on certain psychiatric scale) were done for the assessment of the correction on FDR-based multiple comparisons to obtain null distribution of the tract lengths. The length of the affected subcomponent is used as a statistical index helping differentiate true results from the false ones caused by misalignment.

## Results

### Patient description

All examined patients were in the stage of reconvalescence after arresting acute psychosis, received antipsychotic treatment in chlorpromazine equivalent doses of 200–300 mg. The clinico-dynamic characteristics of the disease course demonstrate a representativeness of the studied sample and adequacy of the material for solving the problem. Thus, the average length of the prodromal stage was 18.4±6.7 years; average manifestation age — 22.2±5.3 years; average age of the first hospitalization — 24.9±9.0 years; average length of the disease from the prodrome onset — 10.8±7.6 years.

Psychometric indications show a morbid state at the time of examination. BFCRS showed 8.7±7.7 points; PANSS P — 24.6±7.6 points; PANSS N — 28.8±7.9 points; PANSS G — 52.1±10.2 points; PANSS total — 105.4±19.4 points; P1 (delirium) — 4.9±1.8 points; P3 (hallucinations) — 3.4±2.1 points; DSM-5 — 13.7±3.9 points; NSA-4 — 20.6±5.4 points; FAB — 14.5±3.4 points; SAS — 3.0±2.8 points.

The majority of examined patients (63.7%) were disabled (unemployed and invalids) for a long time. Besides, patients of the studied subgroup were characterized by family disadaptation, i.e. they have no need for trustworthy relationships and were leading a lonely lifestyle.

### Correlation analysis between the generalized fractional anisotropy and symptom severity

Having analyzed the correlation of the GFA degree with the degree of symptom severity of various domains, data were obtained on the non-uniform correlation of structural features of the brain pathways with the continual symptomatic heterogeneity: with the increase of the symptom severity, positive correlation spoke of the growth of the anatomical connectivity of the brain regions along the tested tract, whereas negative correlation reflected decrease of such connectivity. Considering specific results of symptom correlation with the tracts, it is necessary to take into account that the detected relationships allow one to talk about associativity or contingency of the detected structural and functional changes of the brain tissues but not about the direct cause-and-effect relations. The results presented below consider statistically significant indicators (p=0.05; FDR) in the tracts (see the Figure and Appendix).

**Figure:**
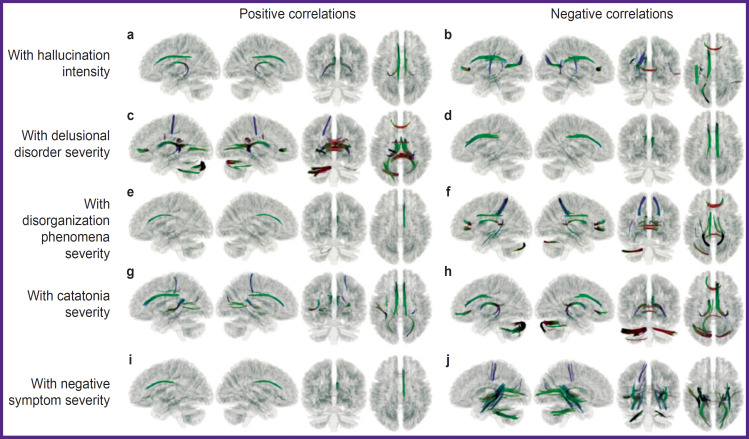
Identified areas of tractographic pathways, for which indicators of generalized fractional anisotropy positively (a, c, e, g, i) and negatively (b, d, f, h, j) correlate with psychometric indicators Color tract encoding is based on the standard RGB (red-green-blue) code, which marks the direction of fiber growth in each point of space: red — left to right, blue — top to bottom, green — front to rear

#### Correlation of structural connectome and hallucination intensity

Positive correlation (Spearman’s correlation coefficient in the voxels changed from 0.40 to 0.58) of the hallucination severity degree and GFA was observed in the following brain pathways (see Figure (a)): left frontal parahippocampal cingulum, left and right parolfactory cingulum, left and right fornix, right frontal parietal cingulum (p=0.05; FDR). Negative correlation (Spearman’s correlation coefficient in the voxels changed from 0.40 to 0.41) of hallucination severity and GFA was observed in the following brain pathways (see Figure (b)): forceps major of corpus callosum, left frontal parietal cingulum, left arcuate fasciculus, left superior longitudinal fasciculus, left posterior thalamic radiation.

#### Correlation of structural connectome and severity of delusional disorder intensity

Positive correlation (Spearman’s correlation coefficient in the voxels changed from 0.40 to 0.43) of the delusion severity was observed in the following brain pathways (see Figure (c)): left and right fornix, forceps major of corpus callosum, forceps minor of corpus callosum, tapetum and body of corpus callosum, left optic radiation, middle cerebellar peduncle, left cerebellum.

Negative correlation (Spearman’s correlation coefficient in the voxels changed from 0.40 to 0.59) of delusion severity and GFA was observed for the following brain pathways (see Figure (d)): right and left frontal parietal cingulum, right parolfactory cingulum, left and right frontal parahippocampal cingulum.

#### Correlation of structural connectome and thought disorganization intensity

Positive correlation (Spearman’s correlation coefficient in the voxels changed from 0.40 to 0.49) of thought disorganization severity and GFA was observed in the following brain pathways (see Figure (e)): right parolfactory cingulum, middle cerebellar peduncle, left inferior cerebellar peduncle.

Negative correlation (Spearman’s correlation coefficient in the voxels changed from 0.40 to 0.50) of delusion severity and GFA was observed in the following brain pathways (see Figure (f)): tapetum and body of corpus callosum, forceps major and minor of corpus callosum, left and right fornix, left frontal parahippocampal cingulum, left cerebellum, left posterior thalamic radiation, left posterior corticostriatal tract, left and right superior longitudinal fasciculus, left superior thalamic radiation.

#### Correlation of structural connectome and catatonia severity

Positive correlation (Spearman’s correlation coefficient in the voxels changed from 0.40 to 0.42) of the brain pathways and catatonia severity was established for the following tracts (Figure (g)): left and right parolfactory cingulum, left frontal parahippocampal cingulum, left and right frontal parietal cingulum, forceps major of corpus callosum, left middle longitudinal fasciculus, right superior thalamic radiation, right medial lemniscus.

Negative correlation (Spearman’s correlation coefficient in the voxels changed from 0.40 to 0.50) of the brain pathway GFA and catatonia severity was registered in the following tracts (Figure (h)): left and right cerebellum, middle cerebellar peduncle, left and right fornix, forceps minor of corpus callosum, right parolfactory cingulum, right frontal parahippocampal cingulum, right frontal parietal cingulum.

#### Correlation of structural connectivity and negative symptom severity

Positive correlation (Spearman’s correlation coefficient in the voxels changed from 0.40 to 0.53) of negative symptom severity and GFA was observed for the following brain pathways (Figure (i)): right frontal parietal cingulum, right frontal parahippocampal cingulum, right parolfactory cingulum, forceps minor of corpus callosum.

Negative correlation (Spearman’s correlation coefficient in the voxels changed from 0.40 to 0.65) of brain pathway GFA and negative symptom severity was fixed in the following tracts (Figure (j)): tapetum of corpus callosum, forceps major of corpus callosum, middle cerebellar peduncle, left medial longitudinal fasciculus, left and right inferior fronto-occipital fasciculus, right parahippocampal parietal cingulum, left inferior longitudinal fasciculus, left and right fornix, left and right posterior corticostriatal tract.

## Discussion

###  

#### Structural connectome and domain of hallucination symptom

We have defined that with the increase of hallucination severity the following changes are observed in a direct relationship:

structural connectivity increases in the brain fornix tracts and cingulum tracts connecting frontal regions with parahippocampal, parietal, and olfactory compartments;
structural connectivity decreases in the tracts of corpus callosum body in parietal and anterior departments connecting lateral and medial areas; in the tracts of the left superior longitudinal fasciculus connecting frontal, parietal, occipital, and temporal regions; in the region of posterior thalamic radiation providing sensory-motor functions; and what was expected, the connectivity reduces along the left arcuate fasciculus between the Broca’s and Wernicke’s speech zones.

Complementary to the obtained results, it is worth noting that data on positive correlation of hallucination severity with alterations in the uncinated fasciculus tracts and left corticospinal tract were reported previously in the work [20].

The observed alterations in the architecture of the brain pathways are supposed to involve changes in the functional connectome. Thus, in patients with non-verbal hallucinations, hyperactivity of the superior temporal gyrus and hypoactivity of prefrontal lobe and anterior cingulum are observed, which correlates with GFA decrease in the left temporo-parietal junction [31]. In chronic persistence of auditory hallucinations, there was noted weakening of the connectivity function and activity in temporal, cingulate, premotor, cerebellar, and subcortical compartments involved in the inner speech and verbal imagination [32], as well as the decrease of the FA coefficient in the zones of hippocampus, splenium, and genu of corpus collossum [33].

#### Structural connectome and domain of delusional disorder symptom

Delusion is an extreme distortion of cognitive processes such as evaluation of reward and evaluation of prediction, when impairment of structural connectivity between prefrontal, limbic, paralimbic, and sensory regions responsible for realization of the obtained social and emotional experience and adequacy of the analysis of what is happening, serves as a pathomorphological substrate [34]. Previously, data were obtained on positive correlation of paranoid delusion and a low FA level in arcuate fasciculus [35], corpus callosum, superior longitudinal fasciculus, and cingulate fasciculus [36]. We have found that there is negative correlation between delusion severity (based on psychiatric examination, it is manifested itself in the qualitative indicator of transition from persecutory to fantastic delusion) and the GFA indicator in the left and right cingulate tracts. We also observed positive correlation with GFA of the corpus callosum and left optic radiation tracts, which were mentioned in a number of investigations of the first psychotic episode of schizophrenia [37, 38]. The appearance of the fantastic, freakish fable is associated with the GFA increase in the tracts of fornix and middle cerebellar peduncles. This result suggests involvement of hippocampus and its structure (processes of memorizing and reactivation of memory traces) and cerebellum structures (autonomic behavior) in the processes of formation and manifestation of delusional disorders.

#### Structural connectome and domain of thought disorganization

In the period of acute psychosis, impairment of purposefulness of thinking processes and their rupture up to verbigeration manifestations along with difficulties in movement coordination and misunderstanding of simple instructions, and even appearance of proboscis symptom are observed. Although this phenomenon is dynamic, it is prognostically important, determining the risk of unfavorable outcome [23]. Disorganization of thinking has been established to be associated with functional neuroimaging defined-dysfunction of brain neural networks, including frontal compartments, auditory cortex, visual system, parietal compartments, limbic system, hippocampal pathways, regions of cingulate gyrus, and so on [39]. In our study we showed positive correlation between thinking impairment and the GFA indicators of the right parolfactory cingulum, tracts of the left middle and inferior cerebellar peduncles; negative correlation was shown to be with the GFA indicator of corpus callosum, brain fornix, tract of the superior longitudinal fasciculus. It is also worth mentioning that correlation analysis helped identify interhemispheric asymmetry, expressed in the changes in GFA of the cingulate tract, thalamic tract, cerebellum, and corticostriatal tract, replicating the results of several studies [39]. As in the previous cases, the data obtained reflect systemic alterations in the brain pathways involving the connectivity of the network regions responsible for executive functions, emotional sphere, memory processes, motor provision, attention, and decision-making as well as autonomic programs of execution.

#### Structural connectome and negative symptom domain

The search for neurobiological markers of negative disorders is one of the priority tasks in current investigations due to the actually low curability rate and direct impact on functional outcome [40, 41]. Negative disorders may be considered as a reflection of neurodegenerative processes and loss of psychic functions in the form of narrowing of the emotional expressivity range and weakening of volitional qualities for feeling plenitude of life and self-realization [42]. One of the works fulfilled in the paradigm of the dimensional approach has demonstrated positive correlation of negative symptom severity with the disturbances of the right anterior thalamic radiation tracts and negative correlation with the right ventral cingulate fasciculus [20]. The data obtained in our study widen the notion on the correlation of structural connectome and intensity of negative symptoms in schizophrenia. Deeper negative disorders are associated with the GFA increase in the right cingulum tracts and the tracts of the corpus callosum and with the GFA decrease not only in the cerebellar tracts and cingulum tract (obtained in the similar work [20]) but in the tracts of the fornix, corpus callosum, left and right corticostriatal tracts, right temporoparietal tract, which provides a ventral optic stream (“Who” function). Clinically important is identification of negative correlation of the negative symptoms and structural alterations in the left posterior compartment of the corticostriatal tract engaged in the volitional regulation of the motor activity, categorization of the objects, and adequate perception of emotions [43].

#### Alterations of structural connectome and catatonia

One of the key pathophysiological processes in catatonia is impaired control of motor acts, emotion expression, and regulation of behavior due to abnormality of horizontal modulation, i.e. prefrontal-orbitofrontal-parietal chain, and vertical modulation, i.e. connecting the compartments of the anterior brain with the deep structures [44]. According to the results of the study [45], it is due to the structural and functional aberrations of the dorsolateral, prefrontal and orbitofrontal cortex that the imbalance in the work of the connectome of the regulating and controlling anterior brain compartments with amygdaloid body, hippocampus, thalamus takes place in catatonia. Characteristic manifestations of catatonia correlate with the dysfunction of the lateral orbitofrontal cortex, while catatonic symptom heterogeneity is determined by the differences in the degree of the damage or dysfunction of some links of the mentioned systems [45]. We have found that catatonia severity correlates with the GFA increase in the cingulate tracts, forceps major of corpus callosum, left middle longitudinal fasciculus, superior thalamic radiation, and right medial lemniscus. Inverse correlation was found between symptom severity and GFA in the tracts of cerebellum, fornix, forceps minor of corpus callosum, tracts of middle cerebellar peduncle, and cingulate tract.

#### Alterations of structural connectome and multiformity of different symptom domain severity

The data obtained by us have shown that alterations of some tracts of the brain white matter are associated with the severity of several symptom domains at once. Thus, changes in the tracts of the right frontal-parietal and parolfactory cingulum correlate with the severity of hallucinations, negative and catatonic symptomatology. The anterior cingulate gyrus is known to be linked with formation of subjective assessments of individual emotional experience and control of executive functions. Alterations in the cingulum connectivity are especially evident in empathy and cognitive regulation of emotions [46]. At the same time, the posterior cingulate gyrus is connected with integration of information into the structure of the individual experience involving the processes of attention, working/long-term memory [36]. Changes in the tracts of the left frontal parahippocampal cingulum correlate negatively with severity of hallucination and delusion, whereas changes in the right frontal parahippocampal cingulum — with the severity of delusion and catatonia. In case of severe hallucinations, delusional disorders, and disorganization, the most significant alterations are seen in the tracts of the left and right fornix. Fornix, as part of limbic system and the main output tract of hippocampus, is engaged in the work of the episodic memory connected with encoding and retrieval of the spatiotemporal patterns and also in the processes of the long-term memory retrieval [47].

Significant changes in the pathways of the corpus callosum, playing a key role in the information interhemispheric interactions, correlated with the prominence of catatonic symptom severity and negative symptomatology. The development of catatonia with severe disorganization phenomena was additionally linked with changes in the connectivity of the sensory-motor system tracts (left and right superior thalamic radiation).

The results obtained in the process of the present study match partly the data published previously by Bopp et al. [20], and complement the information on a set of the altered tracts in the structural connectome related to the severity of psychopathological dimensions. Causes of structural connectome alterations require further exploration. Our results may be explained by various models: the character of neurogenesis in the brain structures, the level of functional activity of various neurotransmitter neuromediator systems involved in psychoses [48], and so on. It should be taken into consideration that changes may have a more complicated form of causal relationships and develop due to the interaction of multiple factors participating in the psychosis pathogenesis. For example, there is a controversial assumption on a combined effect of neurotropic microorganisms [49].

Functional and structural reorganization of the brain neural networks in schizophrenia occurs in ontogenesis and has presumably specific profile. This profile is determined by the dynamics of maturation of the brain neural network structures [50], on the one hand, and consequent loss of the brain grey matter, on the other [51], consistently affecting parietal, frontal cortex and temporal regions. These generalized changes are combined with formation of individual experience in the process of subject interaction with the environment and influence thereby the formation of functional systems of the body. At the level of brain structural organization, this is reflected in the non-linear dependence of the brain region connectivity strength on the intensity of disease symptoms [52]. At the behavioral level, this dynamics of morphometric and tractographic changes is reflected in the complex of ontogenic symptoms involving impairment of integrative image perception, cognitive control function, formation of anticipatory model of behavior, social disadaptation, and others. The data obtained allow us to suppose that at this stage of psychiatry development, it is important to create objective assessment of schizophrenia, the basis of which will be structural and functional architectures of patient brain neural networks as a substrate for realization of psychic functions. Understanding disorders in the brain functional systems will give the possibility to develop individual restoration programs at the next stage.

#### Strong sides of the study

Thorough examination of the nosologically uniform group of patients suffered their first psychotic episode of schizophrenia has been conducted. An important aspect is identifying continual heterogeneity of the main psychopathological domain severity: hallucinations, delusion, disorganization of thinking, catatonia, and negative symptoms. This made it possible not only replicate the results of the other studies performed in the similar design but also to receive new data on the brain tracts, the changes in which correlate with symptomatology.

#### Limitations of the work

Concerning the patients of the examined sample, it can be pointed out that in the clinical aspect (from the point of view of the diagnosis) we speak about a uniform group with the symptoms that are continually heterogeneous in severity. However, the conducted therapy has not been analyzed in detail as well as the characteristics of the period preceding the disease, including perinatal pathology, childhood disorders, and so on, which can potentially affect the morphofunctional brain characteristics.

## Conclusion

During our study, we used a dimensional approach of clinical diagnosis, taking into account continual severity heterogeneity of the main symptoms in the group of patients with schizophrenia. New data have been obtained on the possible differential involvement of the brain structures in the pathogenesis of diverse schizophrenia manifestations such as hallucinations, delusion, disorganization phenomena, and negative disorders.

## APPENDIX

**Table:** Correlation of structural connectome and schizophrenia symptom intensity (p=0.05; false discovery rate or expected proportion of erroneous discoveries)

Symptom	Positive correlation	Negative correlation
Hallucinations	Left frontal parahippocampal cingulum Left and right parolfactory cingulum Left and right fornix Right frontal parietal cingulum	Forceps major of corpus callosum Left frontal parietal cingulum Left arcuate fasciculus Left superior longitudinal fasciculus Left posterior thalamic radiation
Delusional disorder	Left and right fornix Forceps major of corpus callosum Forceps minor of corpus callosum Tapetum and body of corpus callosum Left optic radiation Middle cerebellar peduncle Left cerebellum	Left and right frontal parietal cingulum Right parolfactory cingulum Left and right frontal parahippocampal cingulum
Disorganized thinking	Right parolfactory cingulum Middle cerebellar peduncle Left inferior cerebellar peduncle	Tapetum and body of corpus callosum Forceps major and minor of corpus callosum Left and right fornix Left frontal parahippocampal cingulum Left cerebellum Left posterior thalamic radiation Left posterior corticostriatal tract Left and right superior longitudinal fasciculus Left superior thalamic radiation
Catatonia	Left and right parolfactory cingulum Left frontal parahippocampal cingulum Left and right frontal parietal cingulum Forceps major of corpus callosum Left middle longitudinal fasciculus Right superior thalamic radiation Right medial lemniscus	Left and right cerebellum Middle cerebellar peduncle Left and right fornix Forceps minor of corpus callosum Right parolfactory cingulum Right frontal parahippocampal cingulum Right frontal parietal cingulum
Negative symptomatology	Right frontal parietal cingulum Right frontal parahippocampal cingulum Right parolfactory cingulum Forceps minor of corpus callosum	Tapetum of corpus callosum Forceps major and minor of corpus callosum Middle cerebellar peduncle Left middle longitudinal fasciculus Left and right inferior fronto-occipital fasciculus Right parahippocampal parietal cingulum Left inferior longitudinal fasciculus Left and right fornix and left and right posterior corticostriatal tract

## References

[1] Vitolo E., Tatu M.K., Pignolo C., Cauda F., Costa T., Ando’ A., Zennaro A (2017). White matter and schizophrenia: a meta-analysis of voxel-based morphometry and diffusion tensor imaging studies.. Psychiatry Res Neuroimaging.

[2] Honea R., Crow T.J., Passingham D., Mackay C.E (2005). Regional deficits in brain volume in schizophrenia: a meta-analysis of voxel-based morphometry studies.. Am J Psychiatry.

[3] Podwalski P., Szczygieł K., Tyburski E., Sagan L., Misiak B., Samochowiec J (2021). Magnetic resonance diffusion tensor imaging in psychiatry: a narrative review of its potential role in diagnosis.. Pharmacol Rep.

[4] Demjaha A., Morgan K., Morgan C., Landau S., Dean K., Reichenberg A., Sham P., Fearon P., Hutchinson G., Jones P.B., Murray R.M., Dazzan P (2009). Combining dimensional and categorical representation of psychosis: the way forward for DSM-V and ICD-11?. Psychol Med.

[5] Crow T.J (1985). The two-syndrome concept: origins and current status.. Schizophr Bull.

[6] Liddle P.F., Barnes T.R., Morris D., Haque S (1989). Three syndromes in chronic schizophrenia.. Br J Psychiatry Suppl.

[7] Lindenmayer J.P., Bernstein-Hyman R., Grochowski S., Bark N (1995). Psychopathology of schizophrenia: initial validation of a 5-factor model.. Psychopathology.

[8] Lehman A.F (1999). Improving treatment for persons with schizophrenia.. Psychiatr Q.

[9] Mucci A., Galderisi S., Amodio A., Dierks T, Galderisi S., DeLisi L., Borgwardt S (2019). Neuroimaging and psychopathological domains.. Neuroimaging of schizophrenia and other primary psychotic disorders..

[10] Wheeler A.L., Voineskos A.N (2014). A review of structural neuroimaging in schizophrenia: from connectivity to connectomics.. Front Hum Neurosci.

[11] Zhang F., Daducci A., He Y., Schiavi S., Seguin C., Smith R.E., Yeh C.H., Zhao T., O’Donnell L.J (2022). Quantitative mapping of the brain’s structural connectivity using diffusion MRI tractography: a review.. Neuroimage.

[12] Kraguljac N.V., Lahti A.C (2021). Neuroimaging as a window into the pathophysiological mechanisms of schizophrenia.. Front Psychiatry.

[13] Chan W.Y., Yang G.L., Chia M.Y., Lau I.Y., Sitoh Y.Y., Nowinski W.L., Sim K (2010). White matter abnormalities in first-episode schizophrenia: a combined structural MRI and DTI study.. Schizophr Res.

[14] Alderson-Day B., McCarthy-Jones S., Fernyhough C (2015). Hearing voices in the resting brain: a review of intrinsic functional connectivity research on auditory verbal hallucinations.. Neurosci Biobehav Rev.

[15] Kubera K.M., Rashidi M., Schmitgen M.M., Barth A., Hirjak D., Sambataro F., Calhoun V.D., Wolf R.C (2019). Structure/function interrelationships in patients with schizophrenia who have persistent auditory verbal hallucinations: a multimodal MRI study using parallel ICA.. Prog Neuropsychopharmacol Biol Psychiatry.

[16] Rowland L.M., Spieker E., Holcomb H.H (2009). A review of diffusion tensor imaging in schizophrenia.. Clinical Schizophrenia & Related Psychoses.

[17] Hugdahl K (2015). Auditory hallucinations: A review of the ERC “VOICE’ project.. World J Psychiatry.

[18] Padmanabhan J.L., Tandon N., Haller C.S., Mathew I.T., Eack S.M., Clementz B.A., Pearlson G.D., Sweeney J.A., Tamminga C.A., Keshavan M.S (2015). Correlations between brain structure and symptom dimensions of psychosis in schizophrenia, schizoaffective, and psychotic bipolar I disorders.. Schizophr Bull.

[19] McKnight R., Price J., Geddes J (2019). Schizophrenia and related psychotic disorders.. Psychiatry..

[20] Bopp M.H.A., Zöllner R., Jansen A., Dietsche B., Krug A., Kircher T.T.J (2017). White matter integrity and symptom dimensions of schizophrenia: a diffusion tensor imaging study.. Schizophr Res.

[21] Thomas P., Mathur P., Gottesman I.I., Nagpal R., Nimgaonkar V.L., Deshpande S.N (2007). Correlates of hallucinations in schizophrenia: a cross-cultural evaluation.. Schizophr Res.

[22] Jablensky A., Sartorius N., Ernberg G., Anker M., Korten A., Cooper J.E., Day R., Bertelsen A (1992). Schizophrenia: manifestations, incidence and course in different cultures. A World Health Organization ten-country study.. Psychol Med Monogr Suppl.

[23] Roche E., Creed L., MacMahon D., Brennan D., Clarke M (2015). The epidemiology and associated phenomenology of formal thought disorder: a systematic review.. Schizophr Bull.

[24] Solmi M., Pigato G.G., Roiter B., Guaglianone A., Martini L., Fornaro M., Monaco F., Carvalho A.F., Stubbs B., Veronese N., Correll C.U (2018). Prevalence of catatonia and its moderators in clinical samples: results from a meta-analysis and meta-regression analysis.. Schizophr Bull.

[25] Aandi Subramaniyam B., Muliyala K.P., Suchandra H.H., Reddi V.S.K (2020). Diagnosing catatonia and its dimensions: cluster analysis and factor solution using the Bush Francis Catatonia Rating Scale (BFCRS).. Asian J Psychiatr.

[26] Sicras-Mainar A., Maurino J., Ruiz-Beato E., Navarro-Artieda R (2014). Impact of negative symptoms on healthcare resource utilization and associated costs in adult outpatients with schizophrenia: a population-based study.. BMC Psychiatry.

[27] Bobes J., Arango C., Garcia-Garcia M., Rejas J., CLAMORS Study Collaborative Group (2010). Prevalence of negative symptoms in outpatients with schizophrenia spectrum disorders treated with antipsychotics in routine clinical practice: findings from the CLAMORS study.. J Clin Psychiatry.

[28] Yeh F.C., Verstynen T.D., Wang Y., Fernández-Miranda J.C., Tseng W.Y (2013). Deterministic diffusion fiber tracking improved by quantitative anisotropy.. PLoS One.

[29] Yu W., Lv Q., Zhang C., Shen Z., Sun B., Wang Z, Sun B., Salles A (2015). High-angular diffusion MRI in reward-based psychiatric disorders.. Neurosurgical Treatments for Psychiatric Disorders..

[30] Yeh F.C., Badre D., Verstynen T (2016). Connectometry: a statistical approach harnessing the analytical potential of the local connectome.. Neuroimage.

[31] Zhuo C., Fang T., Chen C., Chen M., Sun Y., Ma X., Li R., Tian H., Ping J (2021). Brain imaging features in schizophrenia with co-occurring auditory verbal hallucinations and depressive symptoms — implication for novel therapeutic strategies to alleviate the reciprocal deterioration.. Brain Behav.

[32] Martí-Bonmatí L., Lull J.J., García-Martí G., Aguilar E.J., Moratal-Pérez D., Poyatos C., Robles M., Sanjuán J (2007). Chronic auditory hallucinations in schizophrenic patients: MR analysis of the coincidence between functional and morphologic abnormalities.. Radiology.

[33] Hubl D., Koenig T., Strik W., Federspiel A., Kreis R., Boesch C., Maier S.E., Schroth G., Lovblad K., Dierks T (2004). Pathways that make voices: white matter changes in auditory hallucinations.. Arch Gen Psychiatry.

[34] Rădulescu A.R., Mujica-Parodi L.R (2008). A systems approach to prefrontal-limbic dysregulation in schizophrenia.. Neuropsychobiology.

[35] Abdul-Rahman M.F., Qiu A., Woon P.S., Kuswanto C., Collinson S.L., Sim K (2012). Arcuate fasciculus abnormalities and their relationship with psychotic symptoms in schizophrenia.. PLoS One.

[36] Fitzsimmons J., Schneiderman J.S., Whitford T.J., Swisher T., Niznikiewicz M.A., Pelavin P.E., Terry D.P., Mesholam-Gately R.I., Seidman L.J., Goldstein J.M., Kubicki M (2014). Cingulum bundle diffusivity and delusions of reference in first episode and chronic schizophrenia.. Psychiatry Res.

[37] Whitford T.J., Kubicki M., Pelavin P.E., Lucia D., Schneiderman J.S., Pantelis C., McCarley R.W., Shenton M.E (2015). Cingulum bundle integrity associated with delusions of control in schizophrenia: preliminary evidence from diffusion-tensor tractography.. Schizophr Res.

[38] Braun U., Schaefer A., Betzel R.F., Tost H., Meyer-Lindenberg A., Bassett D.S (2018). From maps to multi-dimensional network mechanisms of mental disorders.. Neuron.

[39] Harvey P.D., Strassnig M (2012). Predicting the severity of everyday functional disability in people with schizophrenia: cognitive deficits, functional capacity, symptoms, and health status.. World Psychiatry.

[40] Galderisi S., Rossi A., Rocca P., Bertolino A., Mucci A., Bucci P., Rucci P., Gibertoni D., Aguglia E., Amore M., Bellomo A., Biondi M., Brugnoli R., Dell’Osso L., De Ronchi D., Di Emidio G., Di Giannantonio M., Fagiolini A., Marchesi C., Monteleone P., Oldani L., Pinna F., Roncone R., Sacchetti E., Santonastaso P., Siracusano A., Vita A., Zeppegno P., Maj M, Italian Network For Research on Psychoses (2014). The influence of illness-related variables, personal resources and context-related factors on real-life functioning of people with schizophrenia.. World Psychiatry.

[41] Galderisi S., Mucci A., Buchanan R.W., Arango C (2018). Negative symptoms of schizophrenia: new developments and unanswered research questions.. Lancet Psychiatry.

[42] de Wit S., Watson P., Harsay H.A., Cohen M.X., van de Vijver I., Ridderinkhof K.R (2012). Corticostriatal connectivity underlies individual differences in the balance between habitual and goal-directed action control.. J Neurosci.

[43] Gansler D.A., McLaughlin N.C., Iguchi L., Jerram M., Moore D.W., Bhadelia R., Fulwiler C (2009). A multivariate approach to aggression and the orbital frontal cortex in psychiatric patients.. Psychiatry Res.

[44] Hirjak D., Rashidi M., Kubera K.M., Northoff G., Fritze S., Schmitgen M.M., Sambataro F., Calhoun V.D., Wolf R.C (2020). Multimodal magnetic resonance imaging data fusion reveals distinct patterns of abnormal brain structure and function in catatonia.. Schizophr Bull.

[45] Palomero-Gallagher N., Hoffstaedter F., Mohlberg H., Eickhoff S.B., Amunts K., Zilles K (2019). Human pregenual anterior cingulate cortex: structural, functional, and connectional heterogeneity.. Cereb Cortex.

[46] Bubb E.J., Metzler-Baddeley C., Aggleton J.P (2018). The cingulum bundle: anatomy, function, and dysfunction.. Neurosci Biobehav Rev.

[47] Benear S.L., Ngo C.T., Olson I.R (2020). Dissecting the fornix in basic memory processes and neuropsychiatric disease: a review.. Brain Connect.

[48] Efimova O., Pavlov K., Kachanovskiy M., Ayupova A., Zorkina Ya., Morozova A., Andreyuk D., Kostyuk G, Velichkovsky B.M., Balaban P.M., Ushakov V.L (2021). Gene expression asymmetry in the human prefrontal cortex.. Advances in cognitive research, artificial intelligence and neuroinformatics. Intercognsci 2020. Advances in Intelligent Systems and Computing, vol 1358..

[49] Gutiérrez-Fernández J., Luna Del Castillo Jde D., Mañanes-González S., Carrillo-Ávila J.A., Gutiérrez B., Cervilla J.A., Sorlózano-Puerto A (2015). Different presence of Chlamydia pneumoniae, herpes simplex virus type 1, human herpes virus 6, and Toxoplasma gondii in schizophrenia: meta-analysis and analytical study.. Neuropsychiatr Dis Treat.

[50] Gogtay N., Giedd J.N., Lusk L., Hayashi K.M., Greenstein D., Vaituzis A.C., Nugent T.F., Herman D.H., Clasen L.S., Toga A.W., Rapoport J.L., Thompson P.M (2004). Dynamic mapping of human cortical development during childhood through early adulthood.. Proc Natl Acad Sci U S A.

[51] Shmukler A.B (2010). Structural and functional inconsistency of different parts of the brain in schizophrenia: the role of integrative perception.. Sotsial’naya i klinicheskaya psikhiatriya.

[52] Mosina L., Ushakov V., Orlov V., Kartashov S., Zakharova N., Kostyuk G, Samsonovich A.V., Liu T (2024). Assessment and correlation of morphometric and tractographic measures of patients diagnosed with schizophrenia.. Biologically Inspired Cognitive Architectures 2023. BICA 2023. Studies in Computational Intelligence, vol 1130..

